# Near-complete response to low-dose ceritinib in recurrent infantile inflammatory myofibroblastic tumour

**DOI:** 10.3332/ecancer.2021.1286

**Published:** 2021-09-13

**Authors:** Abhenil Mittal, Aarushi Gupta, Sameer Rastogi, Adarsh Barwad, Swati Sharma

**Affiliations:** 1 Department of Medical Oncology, Dr Bhim Rao Ambedkar (BRA) Institute Rotary Cancer Hospital, All India Institute of Medical Sciences, New Delhi 110029, India; 2 Department of Radiodiagnosis, ABVIMS and Dr RML Hospital, New Delhi 110001, India; 3 Department of Pathology, All India Institute of Medical Sciences, New Delhi 110029, India

**Keywords:** ceritinib, infant, inflammatory myofibroblastic tumour, low dose

The images published in this article were previously used by the authors in the article: Gupta A, Sharma S, and Mittal A* et al* (2021) **Recurrent infantile inflammatory myofibroblastic tumor of mesentery—case report and review of imaging findings** *Radiol Case Rep* **16**(3) 504–510 [doi:10.1016/j.radcr.2020.12.027].

The authors have copyright of the images; however, this was not referenced.

